# Supplementary material for: Asynchronous magnetic resonance elastography: Shear wave speed reconstruction using noise correlation of incoherent waves
Source: Magn Reson Med. 2022 Oct 27;89(3):990–1001. doi: 10.1002/mrm.29502 (PMC9792433; doi:10.1002/mrm.29502)
Supplement: Supplementary file 1 — DATA S1 MATLAB code used to generate the numerical simulations of Figure 1 [file MRM-89-990-s002.zip › k-Wave/helpfiles/expandMatrix.html]

expandMatrix :: Functions (k-Wave)


# expandMatrix

Enlarge a matrix by extending the edge values.

## Syntax

```
mat_new = expandMatrix(mat, exp_coeff)
mat_new = expandMatrix(mat, exp_coeff, edge_val)
```

## Description

`expandMatrix` enlarges an input matrix by extension of the values at the outer faces of the matrix (endpoints in 1D, outer edges in 2D, outer surfaces in 3D). Alternatively, if an input for `edge_val` is given, all expanded matrix elements will have this value. The values for `exp_coeff` are forced to be real positive integers (or zero). Note, indexing is done inline with other k-Wave functions using `mat(x)` in 1D, `mat(x, y)` in 2D, and `mat(x, y, z)` in 3D.

For example, running

```
mat = magic(3)
expandMatrix(mat, 1)
expandMatrix(mat, [2 0 1 0], 0)
```

will give the outputs

```
ans =

     8     8     1     6     6
     8     8     1     6     6
     3     3     5     7     7
     4     4     9     2     2
     4     4     9     2     2

ans =

     0     0     0     0     0
     0     0     8     1     6
     0     0     3     5     7
     0     0     4     9     2
```

## Inputs

|  |  |
| --- | --- |
| `mat` | the matrix to enlarge |
| `exp_coeff` | the number of elements to add in each dimension, where `exp_coeff = [a]` or `[x_start, x_end]` in 1D  `exp_coeff = [a]` or `[x, y]` or `[x_start, x_end, y_start, y_end]` in 2D  `exp_coeff = [a]` or `[x, y, z]` or `[x_start, x_end, y_start, y_end, z_start, z_end]` in 3D  (here `a` is applied to all dimensions) |

## Optional Inputs

|  |  |
| --- | --- |
| `edge_val` | value to use in the matrix expansion |

## Outputs

|  |  |
| --- | --- |
| `mat_new` | expanded matrix |

## See Also

`resize`
